# Antiretroviral Therapy Helps HIV-Positive Women Navigate Social Expectations for and Clinical Recommendations against Childbearing in Uganda

**DOI:** 10.1155/2014/626120

**Published:** 2014-09-18

**Authors:** Jasmine Kastner, Lynn T. Matthews, Ninsiima Flavia, Francis Bajunirwe, Susan Erikson, Nicole S. Berry, Angela Kaida

**Affiliations:** ^1^Faculty of Health Sciences, Simon Fraser University, Blusson Hall Room 10522, 8888 University Drive, Burnaby, BC, Canada V5A 1S6; ^2^Mbarara University of Science and Technology, Mbarara, Uganda; ^3^Massachusetts General Hospital (MGH), Center for Global Health, Boston, MA 02114, USA; ^4^Division of Infectious Disease, MGH, Boston, MA 02114, USA

## Abstract

Understanding factors that influence pregnancy decision-making and experiences among HIV-positive women is important for developing integrated reproductive health and HIV services. Few studies have examined HIV-positive women's navigation through the social and clinical factors that shape experiences of pregnancy in the context of access to antiretroviral therapy (ART). We conducted 25 semistructured interviews with HIV-positive, pregnant women receiving ART in Mbarara, Uganda in 2011 to explore how access to ART shapes pregnancy experiences. Main themes included: (1) clinical counselling about pregnancy is often dissuasive but focuses on the importance of ART adherence once pregnant; (2) accordingly, women demonstrate knowledge about the role of ART adherence in maintaining maternal health and reducing risks of perinatal HIV transmission; (3) this knowledge contributes to personal optimism about pregnancy and childbearing in the context of HIV; and (4) knowledge about and adherence to ART creates opportunities for HIV-positive women to manage normative community and social expectations of childbearing. Access to ART and knowledge of the accompanying lowered risks of mortality, morbidity, and HIV transmission improved experiences of pregnancy and empowered HIV-positive women to discretely manage conflicting social expectations and clinical recommendations regarding childbearing.

## 1. Introduction

Women of reproductive age in Sub-Saharan Africa are disproportionately affected by HIV/AIDS, accounting for 80% of the nearly 13 million women living with HIV worldwide [1]. HIV-positive women continue to desire their own children [2–7] and become pregnant [5, 8, 9] after knowing their HIV status. Prior to antiretroviral therapy (ART), pregnancy among HIV-positive women presented significant risks of maternal morbidity, mortality, and sexual and perinatal HIV transmission [10–13]. With expanding access to ART, HIV-positive women now have greater opportunity to pursue childbearing goals, with fewer consequences to maternal, partner, and child health [14–16].

Understanding factors that influence pregnancy decision-making and experiences among HIV-positive women is important for developing integrated reproductive health and HIV prevention, treatment, and care services [8, 17], particularly in high-fertility settings with high HIV prevalence. In Uganda, recent studies observe no relationship between access to ART and a quantitative increase in childbearing desires among HIV-positive women [18, 19]; however, far less is understood about the social and clinical factors that shape the experience of pregnancy in the context of ART access.

The primary objective of this qualitative study was to explore how access to ART shapes pregnancy experiences among currently pregnant HIV-positive women in a high HIV prevalence and high fertility setting in southwestern Uganda.

## 2. Methods

### 2.1. Study Setting

Uganda has one of the highest total fertility rates in the world, estimated at six children per woman [20]. HIV prevalence among adults (aged 15–49 years) is estimated at 7.3%, with higher prevalence among women (8.3%) [21].

This study was conducted in Mbarara, a town with a population of 85,000 people located in southwestern Uganda. Study participants were recruited from the HIV clinic within the Mbarara Regional Referral Hospital. The HIV clinic is the region's primary source for comprehensive HIV care services, which includes ART free-of-charge provided through the Ugandan Ministry of Health with support from the President's Emergency Plan for AIDS Relief (PEPFAR), the Global Fund, and the Family Treatment Fund [22].

Over the recruitment period (2011), national antiretroviral treatment guidelines recommended that HIV-positive adults initiate ART at a CD4 cell count below 250 cells/mm^3^ or below 350 cells/mm^3^ for those with tuberculosis, pregnancy, or WHO stage III or IV disease [23].

### 2.2. Study Participants

Women attending the HIV clinic for follow-up care were approached by a clinic nurse to determine eligibility and willingness to participate in the study. Women were eligible to participate in the study if they were HIV-positive, currently receiving ART, pregnant in their 2nd (13–28 weeks) or 3rd (29–40 weeks) trimester, and willing and able to give informed consent for study participation.

### 2.3. Data Collection

Participants were recruited between October and December 2011 via purposive sampling. Upon giving signed informed consent, participants were asked to complete a brief interviewer-administered intake questionnaire to collect participant age, education level, marital status, number of children, date of HIV diagnosis, duration of ART use, HIV status of the father of the current pregnancy, and if HIV positive, whether this partner was taking HIV medication. After completion of the intake questionnaire, a semistructured in-depth interview was conducted in a private setting adjacent to the clinic. Interviews were conducted in Runyankole, the dominant local language. Using an inductive approach, the interview guide included questions aimed at identifying social- and structural-level factors that shaped pregnancy desires and pregnancy experiences of HIV-positive women. On average, each interview lasted one hour. Participants were compensated for costs associated with transportation to the clinic.

Interviews were audio-recorded and detailed notes were taken during the interview process. Both the primary researcher (JK, interviewer) and research assistant (NF, translator) were present during all interviews. The research assistant was fluent in English and Runyankole. All participants spoke Runyankole, and thus questions and answers over the course of the interview were translated between the participant and primary researcher by the research assistant. While there were initial concerns by the research team that participants might feel uncomfortable having both an English speaking interviewer and translator present during the interviews, early interviews revealed this strategy to be conducive to open dialogue and consistent with Mitchell's observation that study participants often prefer to be interviewed by someone outside of the local clinical or community context [24]. This interview method helped to elucidate various sociocultural norms since participants provided in-depth explanations of customs that may have otherwise been assumed to be understood. Immediately after completion of each interview, the primary researcher and research assistant discussed and reviewed interviews together to gather a comprehensive understanding of main themes and observed reactions [25–27].

### 2.4. Data Analysis

Audio-recordings of the interviews were translated into English and transcribed. Transcripts were independently reviewed and coded and emergent themes were discussed by the research team. Thematic analysis and content analysis as described by Berg [28] and Ulin et al. [29] were used to explore initial interpretations formed during data collection and transcript review.

### 2.5. Ethical Considerations

All participants provided voluntary informed consent at study enrolment. Ethical approval for all study procedures was obtained from the Faculty of Medicine Research and Ethics Review Committee and the Institutional Ethics Review Board of Mbarara University of Science and Technology (MUST) (Mbarara, Uganda) and the Research Ethics Board of Simon Fraser University (Burnaby, Canada). Consistent with national guidelines, study clearance was provided by the Uganda National Council of Science and Technology (UNCST) (Uganda).

## 3. Findings

### 3.1. Sample Characteristics

We interviewed 25 HIV-positive pregnant women, 84% of whom were married or cohabiting. Participants' median age was 29 years (interquartile range (IQR) 27–32) and 32% had a secondary school education or higher. All women were on ART: among the 80% of women diagnosed with HIV prior to the current pregnancy, half started ART prior to the current pregnancy and the remainder started ART during it. The remaining 20% of women were diagnosed with HIV and commenced ART during the current pregnancy. Median duration of ART use was 6 months (IQR 3–24). Twenty percent of women were nulliparous, 36% had 1-2 prior live births, and the remaining 44% had three or more prior live births. Twenty-eight percent of women had experienced the death of a child. Average duration of current pregnancy was 7 months (IQR 6–8). Eighty-eight percent of women reported HIV serostatus disclosure to their current partner, 80% of whom had disclosed prior to the current pregnancy. Overall, 64% of the participants' partners were reported to be HIV-positive, 16% were HIV-negative, and 20% of women did not know their partner's HIV-status. Of the 64% of partners who were HIV-positive, 75% were accessing HIV care (Table 1).

### 3.2. Overview of Qualitative Findings

The findings are presented as factors that influenced women's views about pregnancy as an HIV-positive woman. These data suggest four main themes that shape the pregnancy experience: (1) clinical counselling about pregnancy for HIV-positive women is largely dissuasive but focuses on the importance of ART adherence once pregnant; (2) women demonstrate knowledge about the role of ART in reducing risks to maternal health and of perinatal HIV transmission; (3) this knowledge contributes to personal optimism about pregnancy and childbearing in the context of HIV; and (4) access and adherence to ART creates opportunities for HIV-positive women to discretely manage normative social expectations of childbearing. Each theme is discussed further below.

#### 3.2.1. Clinical Counselling about Pregnancy for HIV-Positive Women

Eight out of 23 women (35%) reported speaking to a health care provider about pregnancy prior to or after becoming pregnant. These women explained that counselling about childbearing was largely dissuasive and emphasized the importance of bearing few or no children to protect their health and families.

 They tell us that giving birth now and again reduces your life span so I decided to reduce the number of children I want. That made me want to stop on the two. (*Participant, 31 years old, 2nd pregnancy*)
 I have not talked about pregnancy planning [at the clinic] but we are always told about family planning and not to give birth to many [children] because you may fail to support them [due to health risks associated with being HIV-positive]. (*Participant, 29 years old, 2nd pregnancy*)

While few participants reported conversations about pregnancy with a health care provider that went beyond advice to avoid pregnancy, nearly all had been counseled about the importance of ART adherence to maintain the woman's health and prevent perinatal transmission.

 They tell us we should not give birth when we are infected, how to care for ourselves, positive living, and adherence to medication. (*Participant, 30 years old, 2nd pregnancy*)
 Before starting on ART, I went to the counselor and was told that adhering to ART is up to you. What they can do is provide the medication to decrease viral load hence less chances of transmission and if [you're] lucky the child will be negative. [*⋯*] So the ball is in your court. (*Participant, 24 years old, 1st pregnancy*)

Despite limited formal counselling about pregnancy outside of ART adherence, peer support at the clinic gave participants optimism about pregnancy. In clinic waiting rooms, women reported meeting other healthy, HIV-positive women, who had given birth to healthy children. These encounters inspired HIV-positive and pregnant women to follow clinic guidelines about adhering to medication for their own health and future child.

 I was so scared when I was told I was positive in the beginning but when I came here [to the HIV clinic] and talked to some of the [mothers], they shared with me that they were positive and gave birth to children who are now four years and negative so I got hope. (*Participant, 29 years old, 2nd pregnancy*)

#### 3.2.2. Personal Optimism about Pregnancy in the Context of ART

After learning their HIV-positive serostatus, most women feared the risks of bearing and rearing an HIV-infected child.

 I [was okay with] giving birth before finding out about my [HIV] status but later when I became [aware that I was] positive, that ceased because I thought I would give birth to an infected child. (*Participant, 33 years old, 4th pregnancy*)

However, nearly all women who expressed this fear also reported that access to ART and knowledge of the accompanying lowered health and transmission risks changed the experience of pregnancy and childbirth.

 When I first learnt my [HIV] status I felt stuck. [*⋯*] I went to the hospital and was given Septrin. I was started on ART in September [6-month pregnant]. It gave me hope of giving birth to a negative baby [this time] if I adhere to medication. (*Participant, 29 years old, 6th pregnancy*)

Women expressed that while knowledge of their HIV status lowered their desired number of children and that starting ART did not change their fertility desire, taking ART made them optimistic about their chances of maintaining their health during and after the pregnancy and their ability to care for their children.

 What has changed [now that I am on ART] is I am hopeful that I can give birth to an HIV-negative child [with this pregnancy] but I am not adding more children. (*Participant, 30 years old, 2nd pregnancy*)

#### 3.2.3. Normative Social Expectations and Support for Pregnancy

Participants described that pregnancy decision-making is seldom made at an individual level; rather it is highly influenced by the desires and expectations of partners, families, and the community. Women's individual views about pregnancy are shaped by their perceptions of gendered roles within society. Narratives revealed that normative gender roles of childbearing create a tension for HIV-positive women attempting to balance their own health alongside the social expectation of motherhood.

 Well, some pressures may be there to have children. For example if you are married and [have] no children, your husband will be told you are barren, and then he gets another woman. (*Participant, 27 years old, 1st pregnancy*)
 If you do not have a child, it does not secure your position in the family and your assets will be taken by someone else. (*Participant, 27 years old, 7 births *[*3 children alive*])

For some women, their partners agreed with their wish to stop reproducing after the current pregnancy. These decisions to stop childbearing were linked to a desire to focus on supporting the mother's health and their existing children.

Other women reported that their partners wanted more children, regardless of the woman's preferences. One woman, with two children and pregnant for the fifth time, reported acquiescing to her husband's wish for another child in order to avoid marital conflict.

 My husband insisted and complained but according to my planning I wanted to stop on the two I had. We had family conflicts to a point of failing to stay together so I decided to comply. (*Participant, 28 years old, 5th pregnancy *[*2 children alive*])

Twenty-two (88%) women had disclosed their HIV status to their partners (Table 1). Several women had also disclosed their HIV-positive status to at least one family member. For many, being the second or third generation of family members to live during the HIV/AIDS epidemic means that their families have experienced people living with HIV both with and without treatment. While HIV disclosure to family can be stigmatizing, women explained that some family members gave them support and advice to maintain their health in the context of HIV and pregnancy.

 When I reached home and told my aunties, they were supportive. They counseled me telling me they are there to help and if I take ART I would be okay, [and so would] the baby, so I calmed down. (*Participant, 31 years old, 2nd pregnancy*)
 My mother was not happy [when I told her I was not taking ART] and told me if I did not go for medication she would not care for me when I became sick. (*Participant, 32 years old, 7th pregnancy*)

Similar to the clinical counselling, the family support women received focused on ART adherence to stay healthy during pregnancy and postpartum. Women explained that once they told select family members that they were pregnant, they received reminders about their clinic review dates, financial support, transport money to the clinic, and advice about breastfeeding and maternal health.

However, even while supporting the women and understanding HIV, family members still expected women to give birth to children. This was most apparent when talking to women who had few children.

 The work of the woman is to give birth, so if you have not given birth you have not done anything for him [the husband or the family]. (*Participant, 33 years old, 3rd pregnancy*)
 When I got married I spent two years without a child [*⋯*] [If I did not start giving birth] there would be quarrels or separation. In my family, they would not be happy and say what I did was not right. (*Participant, 40 years old, 5th pregnancy*)

Women explained that being HIV-positive and pregnant puts their health at risk. At the same time, women described that the personal and social risks of rejecting expectations to bear children were greater than the health risks of pregnancy.

#### 3.2.4. Management of Childbearing Expectations and HIV-Related Stigma

Despite improvements in health and everyday life with access to HIV treatment, social stigma related to being HIV-positive and HIV-positive and pregnant is pervasive. Only two women (8%) stated that they had disclosed their status to their village community. Women described disclosure to her social network as something that was not required when on ART. Further, women explained that they used ART as a tool to help fulfill personal and societal expectations of childbearing, without having to expose themselves to the stigma of being HIV-positive and pregnant. One woman explained why she only disclosed her HIV status to her partner:

 I felt if I can remember to take my drugs and come on the appointment date there was no need of telling them [family and community]. Even, when you tell them [your status], instead they start laughing at you [and may not believe you] [*⋯*] I normally see them saying that one is already dead, they are HIV-positive. (*Participant, 33 years old, 3rd pregnancy*)

Women reported that taking ART allowed them to avoid disclosure, discretely proceed with pregnancy, and avoid stigma. One woman stated that:

 If I know my time for medication, I just take it and I do not have to tell them [the community]. (*Participant, 28 years old, 3rd pregnancy*)

Women described that access to ART allows them to choose to disclose their HIV status to supportive family or friends while also managing social expectations, gender roles, and gossip:

 [Before starting ART] I was so weak and had to be supported but now [I] am okay. People had been saying I was positive but after [I started ART] I became healthier and even the gossip stopped. I was so energetic. (*Participant, 30 years old, 4th pregnancy*)

## 4. Discussion

In this qualitative study with pregnant, HIV-infected women accessing care in rural Uganda, women described challenges negotiating their personal reproductive goals in the context of dissuasive pregnancy messages from healthcare providers and social pressures to have children. With the support of peers and families, most women prioritized adherence to ART as a strategy to balance their own health, have an HIV-negative baby, and meet the social and gendered expectations of childbearing. Women's pregnancy views and experiences were thus shaped through a combination of ART treatment, clinic counselling, family, and peer support. These data suggest that among HIV-infected pregnant women in Uganda, views about and desires for pregnancy were not determined by individual choices alone but reflect larger social and clinical expectations.

Although most participants reported knowing their HIV status and many initiated ART prior to the current pregnancy, few women reported talking about pregnancy with their healthcare provider due to perceptions of provider disapproval of HIV-positive women having children. Women reported being primarily advised against childbearing in order to maintain their own health. These findings are consistent with those from other studies in Uganda [3, 30–33] which found that few HIV-positive women consulted healthcare workers about pregnancy due to expected counselling discouraging childbearing. Discussing plans for pregnancy is an opportunity for providers to address periconception sexual transmission risks and help women plan a safer pregnancy. This gap in clinical counselling is important to address when combining sexual and reproductive health programs.

In the absence of formal counselling about pregnancy, we observed that women rely on community resources to navigate the issues of pregnancy and HIV-positive status. In particular, we found that informal discussion among other positive women at the clinic was an important source of support and information, particularly about decreased risk of perinatal HIV transmission when adhering to ART. While the participants in this study (who are all enrolled in HIV treatment and care) represent only a small fraction of the population of HIV-positive women at risk for pregnancy, our findings suggest that discussion about pregnancy experiences is happening among HIV-positive women attending clinic services, and these discussions are contributing to an informal social support network. Similar observations have been reported in studies about the benefits of peer counselling in helping people living with HIV to experience increased well-being and reduced isolation [34–38]. Given that ART use does not eliminate the risks associated with childbearing among HIV-positive women [39], it remains critical that public health and clinic counselling balance risk messaging to create supportive environments to discuss pregnancy while avoiding overstating the effects of ART and inducing false optimism.

Availability of formal counselling for women who choose to conceive is not common, and partially due to competing demands for provider time and limited resources [2, 40]. The priority for HIV care providers is to ensure that patients receive and adhere to ART in order to remain healthy. A recent study from Mbarara District reported that health care providers do not regularly assess fertility goals of men and women living with HIV. A key step to improve comprehensive reproductive health care and outcomes for people living with HIV (PLWH) may be to support health care workers to routinely assess fertility goals of PLWH in order to offer effective contraception to those who do not want to conceive and safer conception counselling to those who want children [41]. As part of this training, HIV prevention messaging must move beyond the overly simplified ABC (abstinence, being faithful, and condom use) approach to HIV prevention in Uganda and integrate evidence-informed strategies that acknowledge the fertility desires of PLWH [42–44].

The high incidence of pregnancy among HIV-positive women both before [6] and after ART initiation [9] and on-going incidence of perinatal and sexual HIV transmission highlight how better integration of HIV and reproductive health programming is a public health imperative to support desired pregnancies and prevent unwanted pregnancies. Studies from other settings have shown increased contraception uptake when family planning services and HIV care are integrated, and support implementation efforts within respective national health plans [45, 46]. However, integration of HIV and reproductive health services and programming remains a challenge at multiple levels. Integrating new policy and protocol into national health plans is arduous, reproductive health and HIV programs and organizations continue to operate separately, and while providing the capacity for health care workers who interact directly with patients would be ideal, training and improving clinic capacity is needed [47, 48]. Yet, opportunities for change may be on the horizon. The Makerere University Joint AIDS Program (MJAP) implemented the prevention of mother to child transmission (PMTCT) option B+ in 2013 and provided training for all providers in Mbarara District [49]. The current focus on PMTCT provides opportunity for further dialogue about the importance of integrating HIV and reproductive health care [50, 51].

Current information from healthcare providers does, however, translate into women's understandings of how ART may help achieve reproductive goals with lowered risks to maternal, partner, and child health. Such knowledge positively impacted women's views about pregnancy. Women were optimistic about the opportunity to maintain their own health such that they could care for their children in the future, reduce perinatal HIV transmission risks, and avoid social stigma. Previous quantitative studies about ART optimism have suggested that ART plays a role in influencing fertility desire among HIV-positive women [6, 52, 53]. Our study contributes to this growing literature by suggesting that ART access increases women's optimism towards and experiences of childbearing.

The majority of women disclosed their HIV status to partners and family members who provided supportive advice about living with HIV and pregnancy. However, disclosure at the community level remained rare and several women expressed fear of HIV-related stigma. This finding is consistent with other recent studies [54–57] reporting that HIV-positive women experience community-level stigma about pregnancy. Adherence to ART and accompanying health improvements helped women discretely manage HIV status disclosure. Stigma linked to gossip or HIV-related physical symptoms was reported to be mitigated when women were able to pursue ART treatment and pregnancy simultaneously. Consistent with previous studies, ART afforded women the opportunity to feel a return to normalcy [58, 59] in relation to motherhood and social roles due to lessened HIV stigma. We found that access to ART empowers women to pursue reproductive roles within their social community [2, 17, 54] while maintaining their health and family dynamics.

Within and beyond the clinic setting, HIV-positive women continue to experience conflicting pressures related to pregnancy [2, 17, 56, 60]. As previous studies have shown [8, 17, 31, 32, 54], social expectations to bear children push women to negotiate their health alongside their reproductive role, and positive HIV status adds yet another dimension to the dilemma. Participants who reported feeling “stuck” between being HIV-positive and trying to maintain their reproductive roles reported new optimism through ART [3, 6, 52, 55]; knowing that they could give birth to an HIV-negative baby [61] and maintain their own health to care for their children gave them hope for the future.

## 5. Limitations 

Our qualitative study was limited to HIV-positive pregnant women in their second and third trimester. Thus, women who participated had likely accepted their pregnancy and may have portrayed more optimistic messages than women in their first trimester. All participants were attending a tertiary-care HIV clinic in southwestern Uganda and cannot be used to generalize to all women in Uganda who are HIV-positive and/or are unaware of their infection or women living elsewhere. Women accessing care at a tertiary level may also be unique in the level of social support and clinic care that they receive.

## 6. Conclusion

Our findings highlight that HIV-positive women's pregnancy experiences are a reflection of and cannot be dissociated from larger familial, communal, and clinical influences. With treatment, HIV-positive women are optimistic that they can lead healthy lives and bear HIV-negative children. Understanding of HIV prevention and treatment within families and communities creates a more receptive environment for women to disclose their HIV status and receive support. Clinic counselling about ART adherence for the health of HIV-positive women and their future children is a good first step in HIV prevention and care. However, public health messaging must target HIV-positive women, healthcare providers, and the broader community to increase awareness of pregnancy experiences among HIV-positive women on ART and how to best support women to achieve reproductive goals, while minimizing risks to maternal, partner, and child health. Such an approach constitutes an important step towards the design of comprehensive reproductive health programming for women living with HIV.

## Figures and Tables

**Table 1:** Characteristics of study sample.

Characteristic	HIV-positive pregnant women (*n* = 25) *N* (%) Median [IQR]
Median age (years)	29 (27–32)
Education level	
Less than secondary school	17 (68%)
Secondary school or higher	8 (32%)
Marital status	
Currently married or living as married	21 (84%)
Not currently married	4 (16%)
Diagnosed with HIV	
During current pregnancy	5 (20%)
Prior to current pregnancy	20 (80%)
Receipt of ART prior to current pregnancy	
Yes	10 (40%)
No	10 (40%)
N/A (diagnosed with HIV during pregnancy)	5 (20%)
Median number of months receiving ART	6 [3–24]
Median gestation of current pregnancy (months)	7 [6–8]
Number of previous live births	
0	5 (20%)
1-2	9 (36%)
3+	11 (44%)
Median number of previous live births	2 (1–4)
Number of living children	
0	6 (24%)
1-2	11 (44%)
3+	8 (32%)
Experienced the death of a child	
Yes	7 (28%)
No	13 (52%)
N/A (nulliparous)	5 (20%)
Disclosed HIV status to partner∗	
Yes	22 (88%)
No	3 (12%)
Disclosed HIV status to partner prior to current pregnancy	
Yes	20 (80%)
No	5 (20%)
Partner's HIV status∗	
HIV-positive	16 (64%)
HIV-negative	4 (16%)
Do not know	5 (20%)
Partner on Medication∗	
Receiving ART	4 (16%)
On Septrin	2 (8%)
On medication (unknown)	7 (28%)
Not on medication	3 (12%)
Partner HIV-status negative or unknown	9 (36%)

*The referent partner is the father of the participant's current pregnancy.
